# SARS-CoV-2 NSP6 reduces autophagosome size and affects viral replication via sigma-1 receptor

**DOI:** 10.1128/jvi.00754-24

**Published:** 2024-10-24

**Authors:** Cuiling Zhang, Qiwei Jiang, Zirui Liu, Nan Li, Zhuo Hao, Gaojie Song, Dapeng Li, Minghua Chen, Lisen Lin, Yan Liu, Xiao Li, Chao Shang, Yiquan Li

**Affiliations:** 1 Changchun Veterinary Research Institute, Chinese Academy of Agricultural Sciences, Changchun, China; 2 Jiangxi Provincial Key Laboratory of Systems Biomedicine, Jiujiang University, Jiujiang, China; 3 Department of Neurosurgery, First Hospital of Jilin University, Changchun, China; 4 Department of Chemistry, Northeastern University, Shenyang, China; 5 Key Laboratory of Jilin Province for Traditional Chinese Medicine Prevention and Treatment of Infectious Diseases, College of Integrative Medicine, Changchun University of Chinese Medicine, Changchun, China; Loyola University Chicago - Health Sciences Campus, Maywood, Illinois, USA

**Keywords:** NSP6, autophagosomes, SIGMAR1, lysosome

## Abstract

Autophagy is a cellular self-defense mechanism by which cells can kill invading pathogenic microorganisms and increase the presentation of components of pathogens as antigens. Contrarily, pathogens can utilize autophagy to enhance their own replication. Severe acute respiratory syndrome coronavirus 2 (SARS-CoV-2) NSP6 can interact with ATPase proton pump component to inhibit lysosomal acidification, which was implicated in the autophagy process. However, research on how SARS-CoV-2 NSP6 affected autophagy, and its impact on virus replication is still lacking. Coronavirus NSP6 has been reported to promote coronavirus replication by limiting autophagosome expansion. However, this finding has not been confirmed in coronavirus disease 2019 (COVID-19). We investigated the effect of NSP6 protein on autophagosomes in different mutant strains of SARS-CoV-2 and revealed that the size of autophagosomes was reduced by NSP6 of the wild-type and Delta variant of SARS-CoV-2. In addition, we found that SARS-CoV-2 NSP6 localized to the lysosome and had an inhibitory effect on the binding of autophagosomes to the lysosome, which blocked the autophagy flux; this may be related to endoplasmic reticulum (ER)-related pathways. We also found that sigma-1 receptor (SIGMAR1) knock out (KO) reversed NSP6-induced autophagosome abnormality and resisted SARS-CoV-2 infection, which responds to the fact that SIGMAR1 is likely to be used as a potential target for the treatment of SARS-CoV-2 infection. In summary, we have provided a preliminary explanation of the effects on autophagy of the SARS-CoV-2 NSP6 protein from the pre-autophagic and late stages, and also found that SIGMAR1 is likely to be used as a potential target for SARS-CoV-2 therapy to develop relevant drugs.

**IMPORTANCE:**

We have provided a preliminary explanation of the effects on autophagy of the severe acute respiratory syndrome coronavirus 2 (SARS-CoV-2) non-structure protein 6 from the pre-autophagic and late stages, and also found that sigma-1 receptor is likely to be used as a potential target for SARS-CoV-2 therapy to develop relevant drugs.

## INTRODUCTION

Autophagy is necessary and beneficial for cells because it can not only remove damaged organelles and prevent accumulation of toxic protein aggregates but also provide biological energy substances required for cell survival and organisms (1). Although autophagy can act as a defense mechanism against environmental changes that cause cellular damage, accelerate material circulation within cells, reshape cells to protect them, excessive autophagy can also lead to active cell death (2). Physiological autophagy plays a beneficial role in various basic cellular mechanisms in different organs through its intracellular catabolic metabolism activities while pathological autophagy affects disease outcomes. Autophagy can also coordinate with the immune system to enhance and regulate many antiviral immune responses (3–5). As an ancient form of antiviral defense mechanism, autophagy plays an important role especially in antiviral defense when other antiviral mechanisms are lacking (6).

In recent years, new mutant viruses have emerged continuously and caused outbreaks apart from recent ones including, severe acute respiratory syndrome coronavirus 2 (SARS-CoV-2), influenza A (H1N1) virus, highly pathogenic avian influenza H5N1 virus, SARS-CoV, and Middle East respiratory syndrome coronavirus (MERS-CoV), etc. Currently, drugs used clinically against viruses mainly include oseltamivir, and literatures have shown that oseltamivir can induce autophagy in H1N1 virus (7). Therefore, understanding and familiarizing ourselves with the mechanism of cell autophagy after viral entry into the body is beneficial for us to better cope with disease outbreaks and epidemics.

SARS-CoV-2 is the main cause of coronavirus disease 2019 (COVID-19) and consists of four structural proteins and 16 non-structural proteins. Structural proteins play an important role in virus-infected host cells, while non-structural proteins participate in viral genetic material replication and host immune response, causing multiple physiological changes in hosts to provide suitable living conditions for viruses. Among them, non-structure protein 6 (NSP6) is a key protein that contributes to the pathogenicity of the virus, which can induce significant cellular activity defects and reduce cell activity by about 40% (8, 9). SARS-CoV-2 NSP6 can inhibit phosphorylation of interferon regulatory factor 3 (IRF3) by binding with TANK-binding kinase 1, antagonizing type I interferon (IFN-I) production. Its ability to inhibit IFN-I production is stronger than that of SARS-CoV and MERS-CoV (10). A recent study found that SARS-CoV-2 NSP6 can interact with ATPase proton pump component (ATP6AP1) to inhibit lysosomal acidification, which induces activation of NLRP3 inflammasome (9). Lysosomes are organelles present in eukaryotic cells that have the ability to digest aging or damaged organelles as well as pathogens substances, namely participating in activation of autophagy, and normal lysosome acidification is crucial for complete process of autophagy. Previous studies have shown that both SARS-CoV and MERS-CoV have capabilities to neutralize lysosomal pH which affects lysosome acidification and weaken lysosomal degradation function (11, 12). Cottam’s research on avian coronaviruses also found that NSP6 can reduce the ability of autophagosomes to transport virus components to lysosomes for degradation, which helps viruses infect host cells (13). Moreover, NSP6 in SARS-CoV-2 variants and other coronaviruses is highly conserved in terms of genetic nucleotide sequence and protein structure, so NSP6 can be considered as an important target for treating SARS-CoV-2.

The sigma-1 receptor (SIGMAR1) is a chaperone receptor that primarily resides at the mitochondria-associated endoplasmic reticulum (ER) membrane and acts as a dynamic pluripotent modulator regulating cellular pathophysiological processes. The SARS-CoV-2 NSP6 protein directly binds to the SIGMAR1 (14).

In this study, we explore the impact of SARS-CoV-2 NSP6 on autophagic bodies during the process of autophagy from the perspective of host autophagic response, providing new ideas for future development of drugs targeting NSP6.

## MATERIALS AND METHODS

### Cell lines, viruses, plasmid, and chemicals

Human non-small cell lung cancer cell A549 were cultured in Roswell Park Memorial Institute (1640, Sigma-Aldrich, R8758), containing 10% fetal bovine serum (FBS, Invitrogen, 10270), 50 U/mL of penicillin and 50 µg/mL of streptomycin. The SIGMAR1-KO A549 cell line was processed from Ubigene Biosciences Co., Ltd. (Guangzhou, China) and maintained in the same growth medium as above. African green monkey kidney cells Vero E6 (ATCC, CRL-1586) was cultured in Dulbecco’s modified Eagle medium (DMEM, Sigma-Aldrich, D5796) containing 10% FBS (Invitrogen, 10270), 50 U/mL of penicillin, and 50 µg/mL of streptomycin.

SARS-CoV-2 HA-NSP6, SARS HA-NSP6, Beta variant HA-NSP6, and Delta variant HA-NSP6 were synthesized by Sangon Biotech (Shanghai, China). The GFP-LC3 plasmid (psetz-gfplc3) was purchased from InvivoGen (San Diego, California, USA). The GFP-mCherry-LC3 plasmid (PPL70001-2a) was purchased from Bioworld Technology (New York City, USA).

Studies with the SARS-CoV-2 wild-type strain BetaCoV/Beijing/IME-BJ01/2020 and Delta variant (CSTR:16698.06.NPRC6.CCPM-8-V-049-2105-6) were conducted in a biosafety level 3 laboratory as described previously (15, 16). All viruses were propagated and titrated using a standard plaque assay in Vero E6 cells.

Rapamycin (HY-10219), E64d (HY-100229), BD1063 (HY-18101A) were purchased from MedChemExpress (Monmouth Junction, New Jersey, USA)

### Antibody

Antibodies against AKT (4685), p-AKT (4060), AMPK (5831), TSC-2 (4308), mTOR (2983), p-mTOR (5536), Raptor (2280), 4E-BP1 (9644), p-4E-BP1 (2855), S6 (2317), p-S6 (4858), ULK1 (8054), p-ULK1 (Ser757) (14202), p-ULK1 (Ser555) (5869), Atg13 (13273), p-Atg13 (26839), Atg5 (2630), Atg16L1 (8089), VPS34 (4263), VPS15 (14580), Beclin1 (3495), Atg14 (96752), p-Atg14 (Ser29) (92340), Rubicon (8465), UVRAG (5320), FIP200 (12436), ATG101 (13492), ATG7 (8558), p62 (16177), TFEB (37785), TFE3 (14779), LAMP1 (9091), LAMP2 (49067), cathepsin B (31718), cathepsin D (69854), SIGMAR1 (61994), and GAPDH (5174) were obtained from Cell Signaling Technology (Danvers, MA, USA). Anti-LC3 antibody (L8918) was obtained from Sigma-Aldrich. Horseradish peroxidase (HRP)-conjugated secondary antibodies (7074 or 7076), Alexa Fluor 647-Conjugated secondary antibodies (4414), and Alexa Fluor 488-Conjugated secondary antibodies (4408) were purchased from Cell Signaling Technology.

### Plasmids and siRNA (small interfering RNA) transfection

A549 and SIGMAR1-KO A549 cells were transfected with 1.5 µg of HA-NSP6, GFP-LC3, or GFP-mCherry-LC3 plasmid using Lipofectamine 3000 reagent (Thermo Fisher Scientific, L3000015), and experiments were conducted 24 h post transfection.

The siRNAs against SIGMAR1 (siG170829033837-1-5) were purchased from RiboBio (Guangzhou, China). Twenty-four hours after seeding, A549 cells were transfected with 50 nM of siRNA using Lipofectamine RNAiMAX reagent (Thermo Fisher Scientific, 13778150) and experiments were conducted 24 h post transfection.

### Transmission electron microscopy (TEM)

Cells were seeded into six-well plates and transfected with SARS-CoV-2 HA-NSP6, SARS HA-NSP6, Beta variant HA-NSP6, or Delta variant HA-NSP6 plasmid 1 day later. After 24 h, cells were collected, lysed, and then fixed with 2.5% glutaraldehyde. Areas containing cells were block mounted and sliced. Ultrathin sections were stained with uranyl acetate and lead citrate before TEM analysis. The number of autophagosomes per cell was calculated and at least 30 cells were included.

### Autophagy change detection

Cells were grown in six-well plates and transfected with SARS-CoV-2 HA-NSP6 plasmid for 24 h. Next, cells were transfected with the plasmid EGFP-LC3. After 48 h, cells were fixed with 4% paraformaldehyde for 15 min, and changes in the green fluorescence of LC3 were observed under a confocal microscope (CARL ZEISS LSM980, Germany).

### Western blotting analysis

Cells were seeded into six-well plates and transfected with SARS-CoV-2 HA-NSP6 plasmid 1 day later. After 24 h, cells were collected and lysed, and protein concentrations were determined using a BCA protein assay kit (Beyotime, Shanghai, China, P0010). The proteins were mixed with loading buffer (Beyotime, Shanghai, China, P0015L) and incubated at 95°C for 10 min, and then equal amounts (25 µg) were electrophoresed, transferred onto a nitrocellulose filter membrane, and then incubated overnight at 4°C with primary antibodies as described previously. After a further incubation with HRP-conjugated secondary antibodies, specific protein band were visualized using Pierce ECL Western Blotting Substrate (Thermo Fisher Scientific, 32106) and an Amersham Imaging 600 system.

### Total proteome workflow

#### Protein extraction

Sample was sonicated 3 min on ice using a high-intensity ultrasonic processor (Scientz) in lysis buffer (8 M urea, 1% protease inhibitor cocktail). The remaining debris was removed by centrifugation at 12,000 *g* at 4°C for 10 min. Finally, the supernatant was collected and the protein concentration was determined with BCA kit according to the manufacturer’s instructions.

#### Trypsin digestion

The sample was slowly added to the final concentration of 20% (m/v) trichloroacetic acid (TCA) to precipitate protein, then vortexed to mix and incubated for 2 h at 4°C. The precipitate was collected by centrifugation at 4,500 *g* for 5 min at 4°C. The precipitated protein was washed with in 200 mM triethylamine borane (TEAB) and ultrasonically dispersed. Trypsin was added at 1:50 trypsin-to-protein mass ratio for the first digestion overnight. The sample was reduced with 5 mM dithiothreitol for 60 min at 37°C and alkylated with 11 mM iodoacetamide for 45 min at room temperature in darkness. Finally, the peptides were desalted by Strata X SPE column.

### Proteomic data mining

The MS/MS data obtained were processed using the MaxQuant search engine (v.1.6.15.0). Tandem mass spectra were searched against the human SwissProt database (20,422 entries) and the reverse decoy database. Trypsin/P was designated as the cleavage enzyme, allowing up to two deletions to be cleaved. The mass tolerance for precursor ions was set at 20 ppm for the first search, 5 ppm for the primary search, and 0.02 Da for fragment ions. Carbamoylmethyl on Cys was designated as a fixed modification, and acetylation at the N-terminus of the protein and oxidation on Met were designated as variable modifications. Data were normalized using MaxQuant 1.5.8.3 software to provide log2 values. These values were entered directly into the statistical computing R-project (v.3.5.1), and the pheatmap function was selected for categorical analysis to generate heatmaps and hierarchical clustering. Pathways associated with differential proteins were identified using Kyoto Encyclopedia of Genes and Genomes (KEGG) pathway analysis.

### Co-localization observation assay

Cells in the logarithmic growth phase were seeded at 1 × 10^5^ cells/well in a 12-well cell culture plate pre-coated with cell slides and cultured at 37°C and 5% CO_2_. Next, cells were transfected with the SARS-CoV-2 NSP6 plasmid for 24 h. Mitochondrial red fluorescent staining kit (BB-44113-1), Golgi BBcellProbe G02 red fluorescent staining kit (BB-44118-1), and endoplasmic reticulum red fluorescent staining kit (BB-441164-1) were purchased from BestBio (Shanghai, China). LysoTracker Red (L7528) was purchased from Thermo Fisher Scientific. Live cell dyes for lysosomes, mitochondria, endoplasmic reticulum, and Golgi were added for staining for 20 min, and samples were fixed with 4% paraformaldehyde for 30 min. After washing, the membranes were blocked with a blocking solution for 30 min. After discarding the blocking solution, the primary antibody solution was added to the 12-well plate, and the plate was gently shaken at 4°C overnight. The primary antibody solution was removed, and cells were washed with PBS. Subsequently, the corresponding secondary antibody solution was added to meet different immunofluorescence co-localization requirements. After cleaning with PBS, the climbing piece was removed and attached to a slide. A nail polish seal was used to avoid drying, and photographs were taken under an inverted fluorescence microscope.

### Cytotoxicity and antiviral activity assays

Cytotoxicity assays were evaluated using the CCK-8 regent (Dojindo Molecular Technologies, Rockville, USA) according to the manufacturer’s instructions. Cells were inoculated in 96-well plates at 7 × 10^3^ cells per well and incubated at 37°C and 5% CO_2_ for 24 h. Thereafter, the medium containing 0, 6.25, 12.5, 25, 50, and 100 µM of the BD1063 was added to the cells and incubated for 2 h. Finally, the medium was removed and 100 µL CCK8 (1 mg/mL in DMEM medium) was added into each well.

Antiviral activity assays were evaluated using the CCK-8 regent. Briefly, BD1063 was added 2 h prior virus treatment. The culture plates were incubated at 37°C in a humidified 5% CO_2_/95% air incubator for 48 h. Following this step, the medium was removed and 100 µL CCK8 (1 mg/mL in DMEM medium) was added into each well and incubated for 3 h at 37°C. The optical density at the dual wave lengths of 450/630 nm was determined using a microplate reader (BIO-TEK EPOCH, USA). The results were transformed to a percentage of the controls. The dose-response curves were plotted using the GraphPad Prism 6 software.

### Crystal violet staining

Cell proliferation was examined using crystal violet staining. Briefly, cells (2 × 10^5^ cells/well) were transferred into 96-well plates and incubated at 37°C, 5% CO_2_ for 24 h. Cells were pre-treated with BD10063. After incubation for 48 h, the cells were stained with crystal violet and fixed with 4% paraformaldehyde for 1 h. Finally, the stained cells were analyzed by microscopy and representative images were captured using a digital camera.

### Measurement of viral titers

Vero E6 cells were seeded in 96-well plates at 7 × 10^3^ cells per well, 50 µL of the BD10063 at different concentrations were added to these cells, and then cells were infected with different coronavirus at a multiplicity of infection (MOI) of 0.08. Supernatant containing the viruses or BD1063 was collected 48 h after transfection, and 10-fold dilutions were added to 96-well plates in a volume of 100 µL/well and at a Vero E6 cell density of 7 × 10^3^ 1 day before. After 2 h of infection, 100 µL DMEM containing 4% fetal bovine serum, 100 IU/mL penicillin, and 100 µg/mL streptomycin were added to a final volume of 200 µL/well. Finally, the cells were incubated at 37°C and 5% CO_2_ for 5 days. Viral titers were calculated as TCID_50_ (median tissue culture infective dose) using the Reed-Muench method.

### Statistical analysis

Statistical analysis was performed using GraphPad software, and the data were processed in the form of (x ± S). Tukey’s test was used or comparisons between two groups; differences were considered statistically significant at *P* < 0.05 or *P* < 0.01.

## RESULTS

### NSP6 activates autophagy

**Fig 1:**
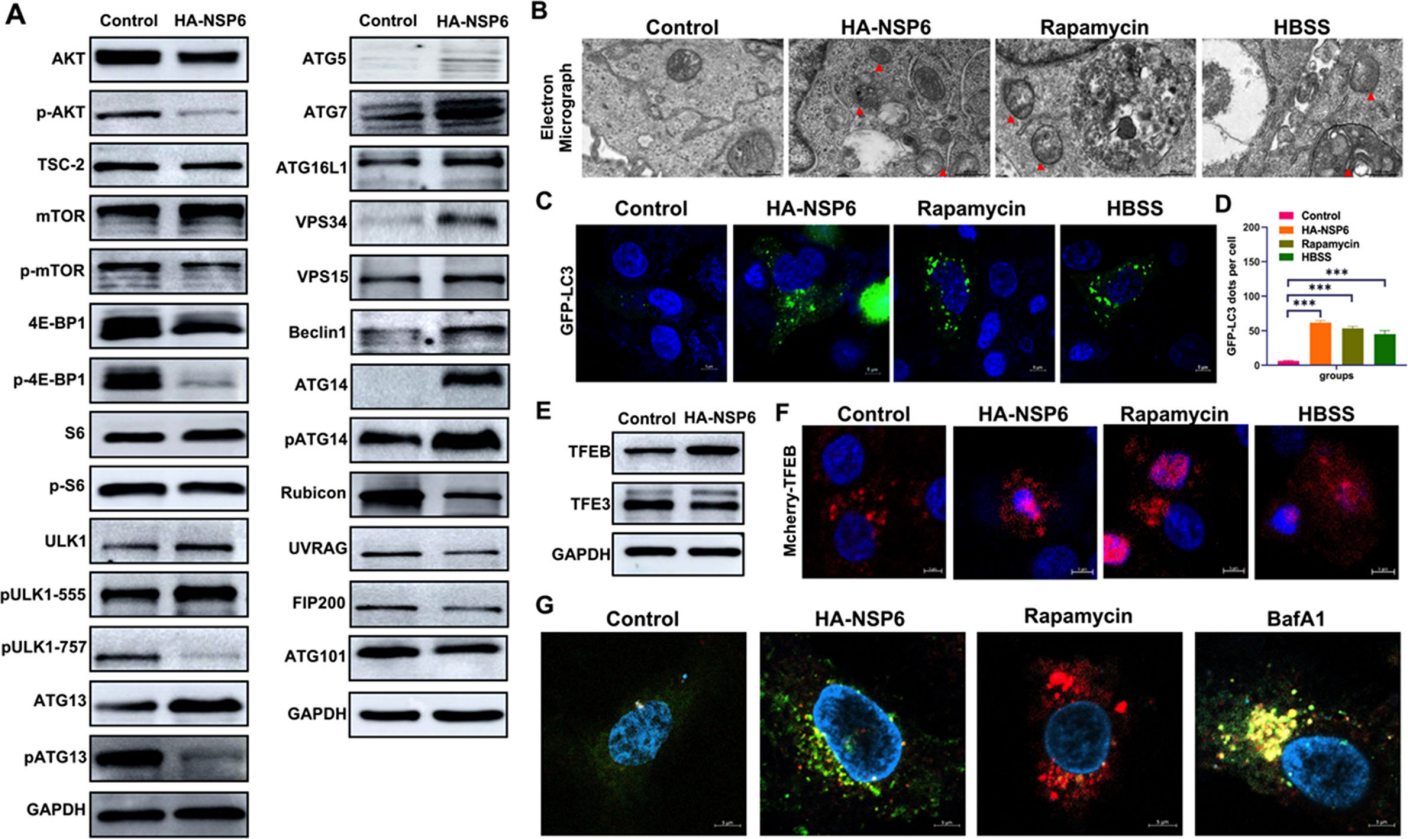
Effects of NSP6 protein on autophagy-related proteins. (**A**) Western blotting showed autophagy-related protein expression in A549 cells with SARS-CoV-2 HA-NSP6 transfection. (**B**) Formation of autophagosomes in A549 cells observed by electron microscopy. Black scale bars = 500 nm. (**C**) Formation of autophagosomes in A549 cells observed by confocal microscopy. White scale bars = 5 µm. (**D**) Calculation of the number of LC3 fluorescent dots. ***P < 0.001. (**E**) Western blotting showed TFEB and TFE3 protein expression in A549 cells with HA-NSP6 transfection. (**F**) Observation of TFEB nucleation phenomenon using confocal microscopy. White scale bars = 5 µm. (**G**) Observation of the production of autophagy and autophagosome using GFP-mCherry-LC3 staining. White scale bars = 5 µm.

We first analyzed the expression of autophagy pathway proteins after NSP6 enters the cell, and found that NSP6 activates the VPS34 complex through the Akt-mTOR-ULK1 pathway, inducing an increase in the expression of Atg5 and Atg16 (Fig. 1A; Fig. S1). Subsequently, electron microscopy observation showed that autophagosomes were formed in the cells treated with NSP6 (Fig. 1B). Transfection with GFP-LC3 plasmid also showed a significant increase in punctate fluorescence of LC3 (Fig. 1C and D). This indicates that NSP6 can activate autophagy in A549 cells. Additionally, transcription factor EB (TFEB) is translocated from cytoplasm to nucleus during autophagy activation and binds to promoter regions of many autophagy-related genes to induce autophagy (17). We found increased expression and translocation of TFEB into the nucleus after treatment with NSP6 (Fig. 1E and F). Meanwhile, transcription factor E3 (TFE3) correlated with increased lysosome numbers and activity, mitochondrial biogenesis autophagy (18). We found reduced expression TFE3 after treatment with NSP6 (Fig. 1E). Combined with observations of autophagosome formation under electron microscopy and fluorescence microscopy, it suggests that NSP6 activates cellular autophagy. In order to further analyze the integrity of autophagy process, we used the activator and inhibitor of autophagy as control, and performed GFP-mCherry-LC3 staining assay. It was found that NSP6 induced the formation of a large number of autophagosomes (yellow fluorescence), which was similar to that of BafA1 (bafilomycin A1) group, while RAPA (rapamycin) group produced a large number of autolysosomes (red fluorescence) (Fig. 1G). The results showed that NSP6 could activate autophagy and form autophagosome, but inhibited the fusion of autophagosome and lysosome.

### Effect of different coronavirus NSP6 proteins on autophagosome size

**Fig 2:**
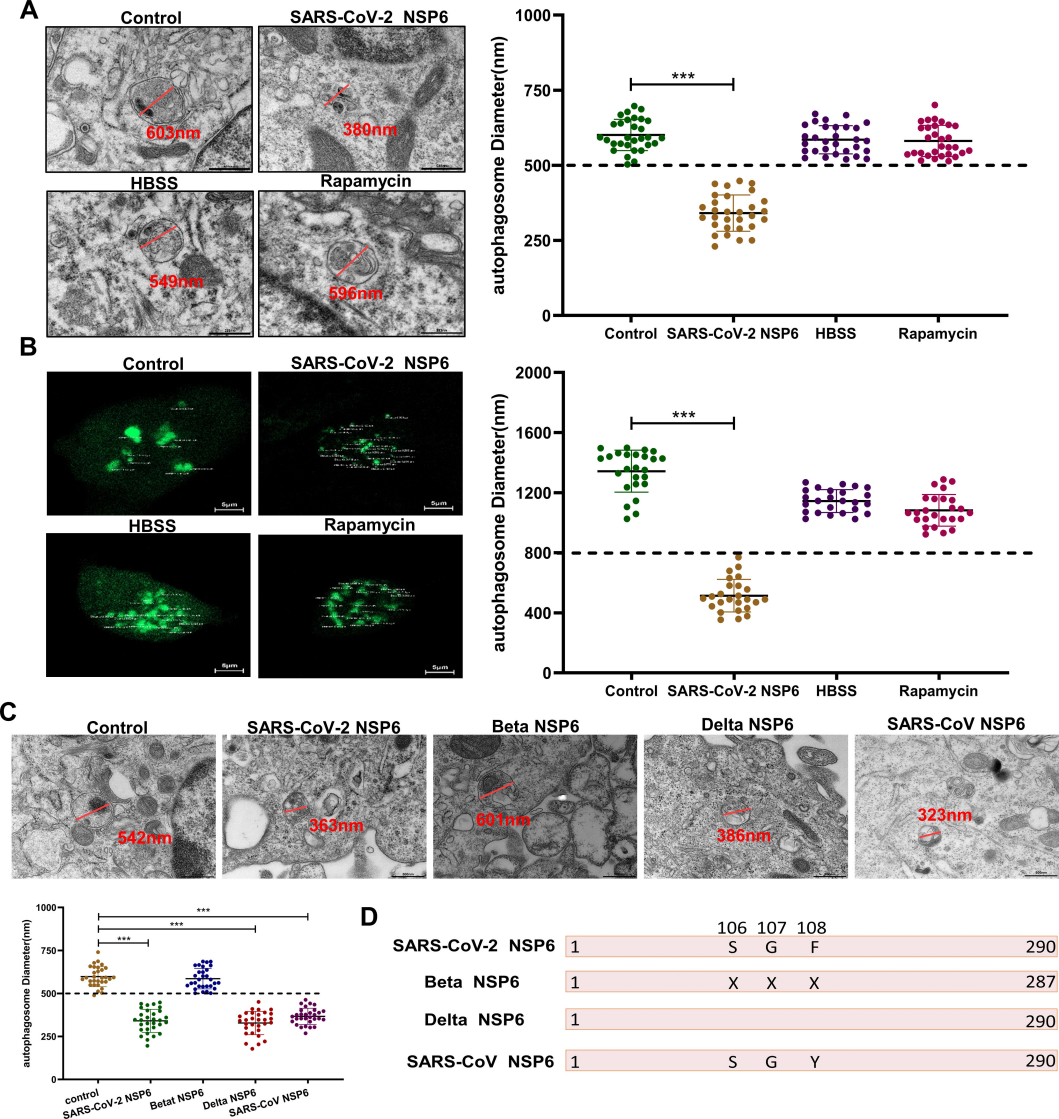
Effects of NSP6 proteins of different virulent strains on changes in autophagosome diameter. (**A**) Changes in the diameter of autophagosomes in A549 cells after NSP6 transfection, rapamycin treatment, or starvation were observed using electron microscopy. Black scale bars = 500 nm. *n* = 30. ***P < 0.001. (**B**) Changes in the diameter of autophagosomes in A549 cells after NSP6 transfection, rapamycin treatment, or starvation were observed using confocal microscopy. White scale bars = 5 µm. *n* = 25. ***P < 0.001. (**C**) Changes in the diameter of autophagosomes in cells after transfection with NSP6 of different SARS-CoV-2 variants and SARS-CoV were observed using electron microscopy. (**D**) Amino acid sequence comparison results of different strains of NSP6.

We investigated the effects of starvation, rapamycin, and SARS-CoV-2 NSP6 on the size of autophagosomes using TEM and laser confocal detection methods. The results showed that although starvation or rapamycin treatment and NSP6 treatment both led to an increase in the number of autophagosomes (Fig. S2), only the diameter of autophagosomes in NSP6 group became smaller (Fig. 2A and B). Next, we transfected NSP6 of SARS-CoV and different SARS-CoV-2 variants into cells to investigate their effects on the size of autophagosomes. TEM images showed that transfection with NSP6 proteins of SARS-CoV and wild-type strain and Delta variant of SARS-CoV-2 significantly reduced the size of autophagosomes. However, no change was observed when using Beta variant strain (Fig. 2C). Finally, by comparative analysis of the amino acid sequences, we found that this phenomenon may be related to the differences in amino acids at positions 106–108 (Fig. 2D).

### NSP6 can affect autophagosome binding to lysosomes

**Fig 3:**
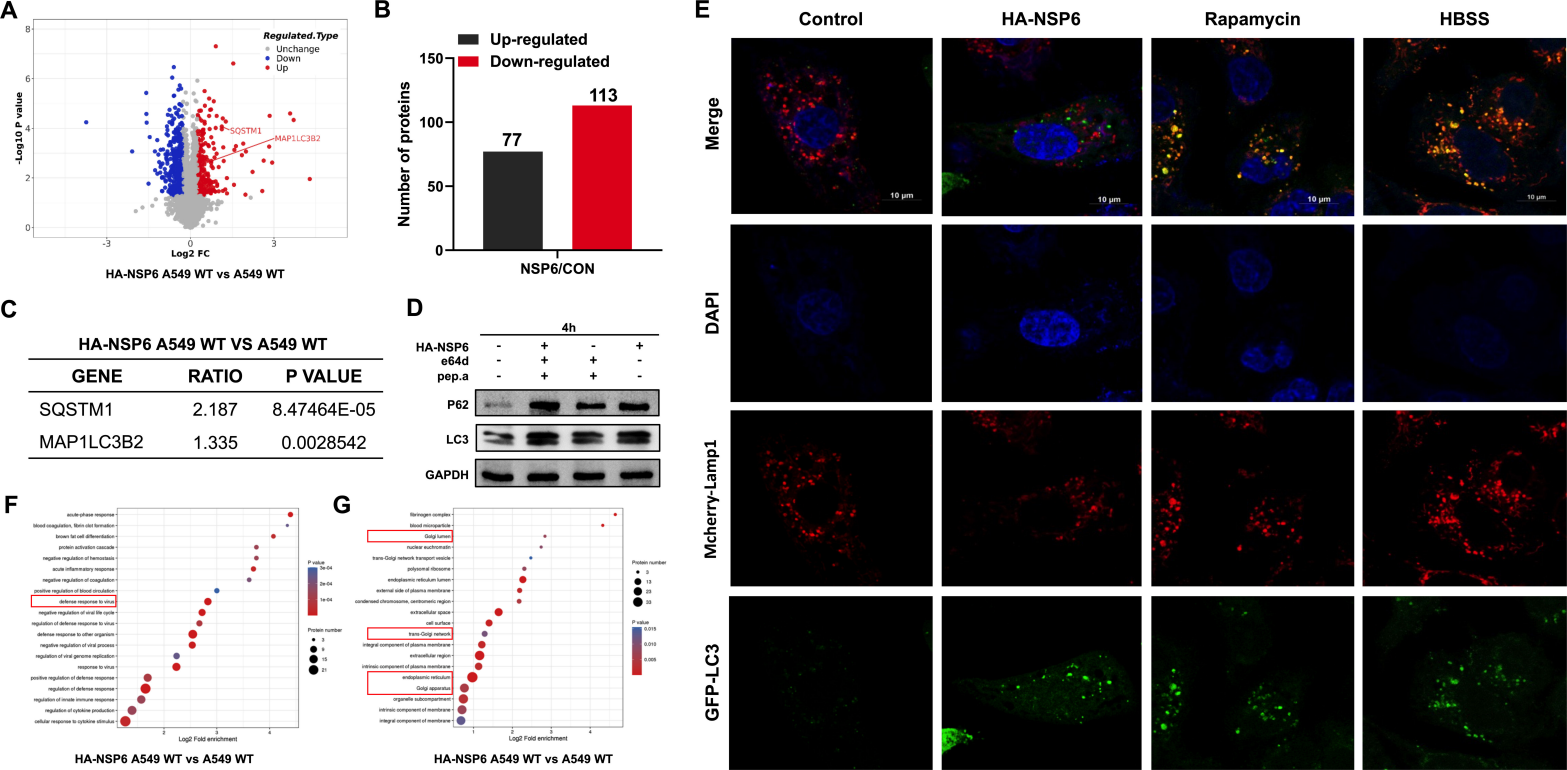
Proteomic analysis of pathways and gene enrichment affected by NSP6. (**A and B**) Proteomic analysis of differential gene expression and number of differential genes. (**C**) Differences in LC3 and P62 in A549 cells after transfection with NSP6 of SARS-CoV-2 wild-type strain. (**D**) Detection of LC3 and P62 expression levels in A549 cells transfected with NSP6 followed by addition of lysosomal protease inhibitors E64d and pepstain A. (**E**) The co-localization of autophagy and lysosome was detected by immunofluorescence experiment. White scale bars = 10 µm. (**F**) Proteomic analysis of differential gene enrichment pathways. (**G**) Proteomic analysis of differential gene-enriched cell structures.

To further elucidate the relationship between NSP6 and autophagy as well as autophagosome size, we transfected HA-NSP6 plasmid into A549 cells and performed proteomic analysis. The results showed upregulation of 77 differentially expressed genes and downregulation of 113 differentially expressed genes (Fig. 3A and B), mainly enriched in virus defense signaling pathways, Golgi membrane, Golgi network, Golgi surface, and endoplasmic reticulum-related pathways (Fig. 3F and G). Differential gene analysis revealed that NSP6 significantly increased expression of LC3 and P62 in A549 cells (Fig. 3C). The increase in P62 indirectly suggests a blockage in autolysosome formation, indicating that NSP6 may affect the fusion of autophagosomes with lysosomes. We also tested the effects of lysosomal protease inhibitor E64d and pepstain A on lysosomal function after transfection with NSP6 proteins in cells. It was found that the expression of P62 was significantly increased when only inhibitors were added, but the expression of P62 was not significantly increased by adding inhibitors after transfection of NSP6 (Fig. 3D), which may be that NSP6 has strongly inhibited the lysosomal function in cells. In addition, we also found that the addition of NSP6 can reduce the number of lysosomes, while LC3 will not co-locate lysosomes (Fig. 3E). These suggest that NSP6 may affect the fusion of autophagosomes with lysosomes by causing damage to lysosomes.

### NSP6 causes lysosomal damage through ER-associated pathways

**Fig 4:**
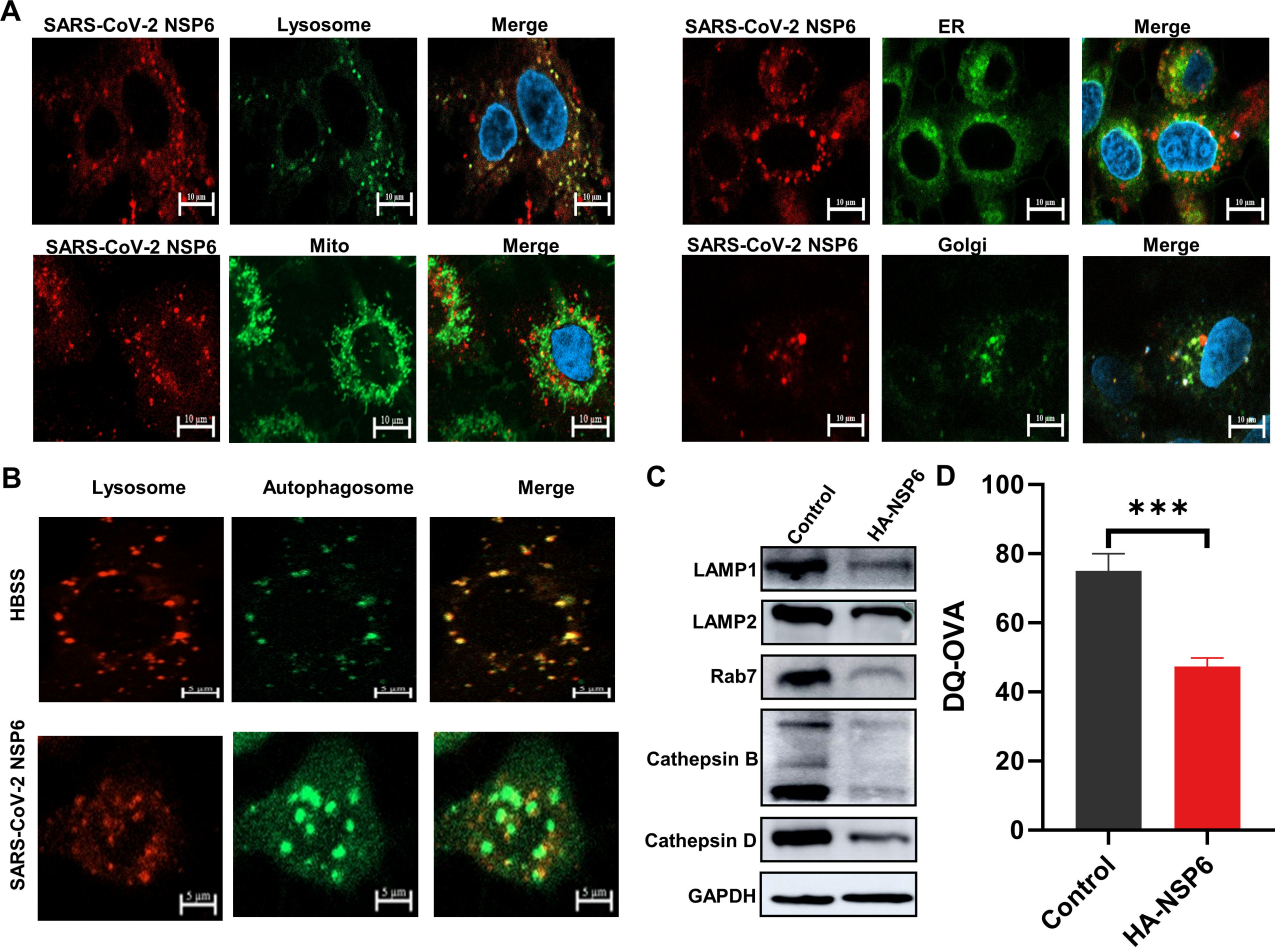
Effects of NSP6 on lysosomes. (**A**) Co-localization of NSP6 with lysosomes and endoplasmic reticulum was observed using confocal microscopy. Red fluorescence refers to NSP6, green fluorescence refers to organelle. White scale bars = 10 µm. (**B**) Observation of lysosomes with or without co-localization with autophagosomes after transfection of A549 cells with NSP6 or starvation treatment. Red fluorescence refers to lysosomes, green fluorescence refers to autophagosomes. White scale bars = 5 µm. (**C**) Western blotting showed lysosome-related protein expression in A549 cells with HA-NSP6 transfection. (**D**) Lysosomal degradation by flow cytometry detection of fluorescence intensity of DQ-OVA. *n* = 5. ***P < 0.001.

By using confocal microscopy, we observed co-localization between NSP6 and lysosomes (Fig. 4A). We speculated whether NSP6 affects lysosomal function as well as the formation of autophago-lysosomes at the late stage of autophagy. Subsequently, we stained for lysosomes and autophagosome markers, and found NSP6 can inhibit fusion between autophagosomes and lysosomes (Fig. 4B). LAMP1 and LAMP2 are components of lysosome membranes, and represent markers for lysosomes. Western blotting showed significant reduction in LAMP1 and LAMP2. Rab7 is a marker for endosomes, which also exhibits a decrease in expression (Fig. 4C). Cathepsin protein family is a group of vesicle enzymes, including cathepsin B and cathepsin D. These proteins mainly exist in lysosomes and participate in the degradation of substances inside autophagic vesicles. Western blotting showed that their expression levels were also significantly reduced (Fig. 4C). In addition, the fluorescence intensity of fluorescent dye-quenched ovalbumin (DQ-OVA) can reflect the degradative capacity of lysosomes. We detected DQ-OVA by flow cytometry and found that the fluorescence intensity decreased, indicating that NSP6 affects lysosomal degradation ability (Fig. 4D). Under confocal microscopy, we also observed that NSP6 has produced co-localization with ER (Fig. 4A), and enrichment of differentially expressed genes associated with the ER pathway was also seen in the proteomics results after transfection with NSP6. This suggests that NSP6 not only causes damage to lysosomes, but may also have some effects on ER.

### SIGMAR1 does not affect NSP6-mediated autophagy activation

**Fig 5:**
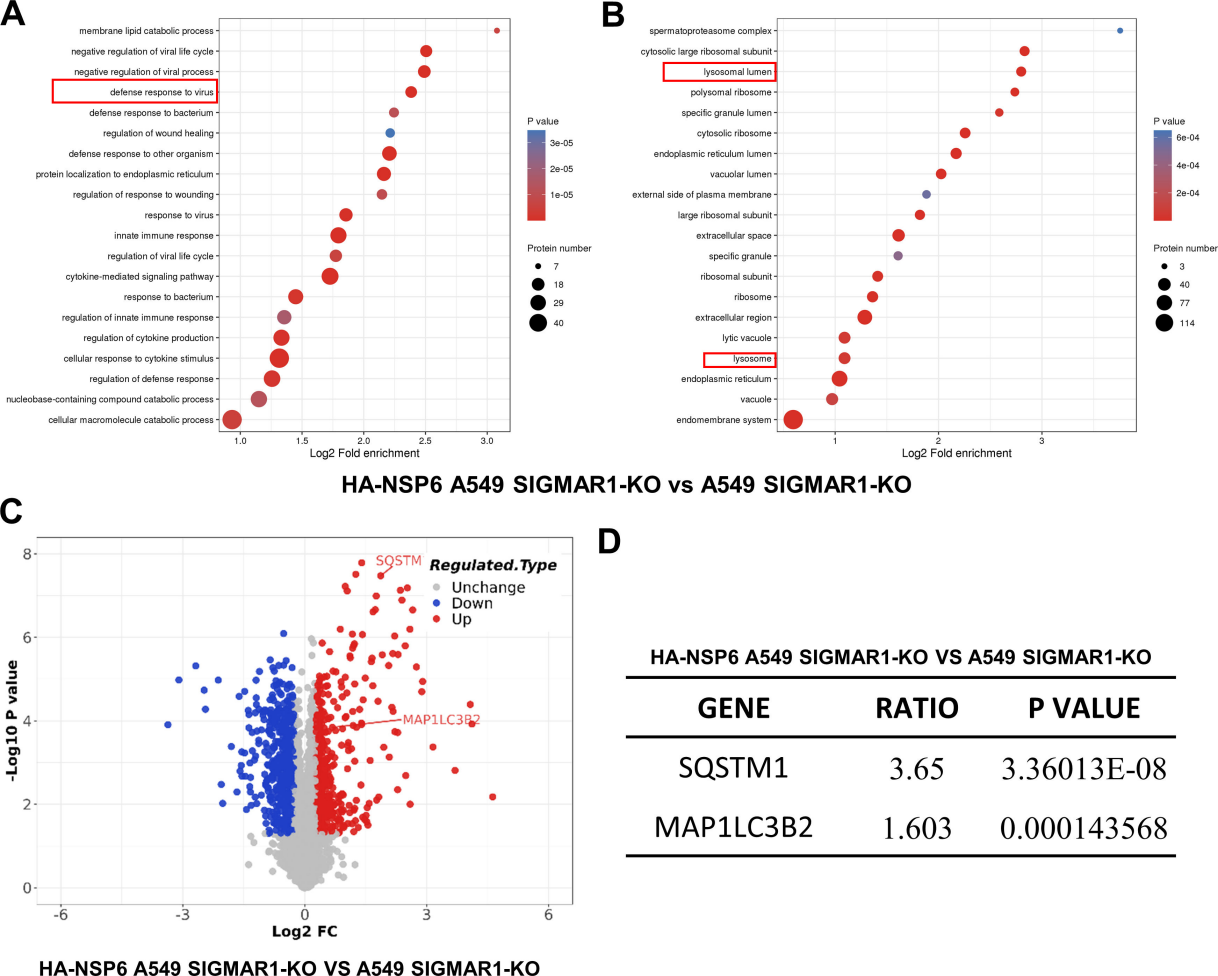
Proteomic analysis of pathways and gene enrichment affected by NSP6 after knockdown of SIGMAR1. (**A**) Proteomic analysis of differential gene enrichment pathways. (**B**) Proteomic analysis of differential gene-enriched cell structures. (**C**) Proteomic analysis of differential gene expression and number of differential genes. (**D**) Differences in LC3 and P62 in SIGMAR1-KO A549 cells after transfection with NSP6.

It has been reported that SARS-CoV-2 can mediate ER stress and induce autophagy, with autophagosome membrane possibly originating from the ER (19, 20). Therefore, we speculated whether the smaller diameter of autolysosomes and the occurrence of incomplete autophagy are related to a specific protein on the surface of the ER membrane. It has been found that SIGMAR1, a unique protein on the surface of the ER membrane, could promote upstream transcription factor TFEB into nucleus and mediate autophagy (21). Therefore, we analyzed whether SIGMAR1 would affect the autophagosome size reduction mediated by NSP6. To clarify the relationship between NSP6 and SIGMAR1, we transfected NSP6-HA plasmid in SIGMAR1-KO A549 cells and performed proteomic analysis. First, we performed principal component analysis (PCA) on A549 WT (wild type) group, HA-NSP6 A549 WT group, HA-NSP6 A549 SIGMAR1 KO group, and A549 SIGMAR1 KO group, and found that all of them were significantly different from the corresponding control group after transfection of NSP6. In addition, there was also a significant difference between the group transfected with NSP6 alone (HA-NSP6 A549 WT group) and the group transfected with NSP6 after knockout of SIGMAR1 (HA-NSP6 A549 SIGMAR1 KO group), suggesting that SIGMAR1 does affect the function of NSP6 (Fig. S3). Subsequently, further analysis of proteomic results showed that the variability of P62 and LC3 in SIGMAR1-KO cells transfected with NSP6-HA did not decrease, but still had significant changes (Fig. 5C and D), indicating that the knockout of SIGMAR1 did not affect the activation of NSP6 on autophagy. Enrichment analysis showed that NSP6 had a significant effect on virus defense and lysosomal pathway in SIGMAR1-KO cells (Fig. 5A and B). This indicated that SIGMAR1 did not affect the autophagy induction of NSP6, but it may be involved in the lysosomal damage and the reduction of autophagosome size caused by NSP6.

### NSP6 induces autophagosome size reduction via SIGMAR1

**Fig 6:**
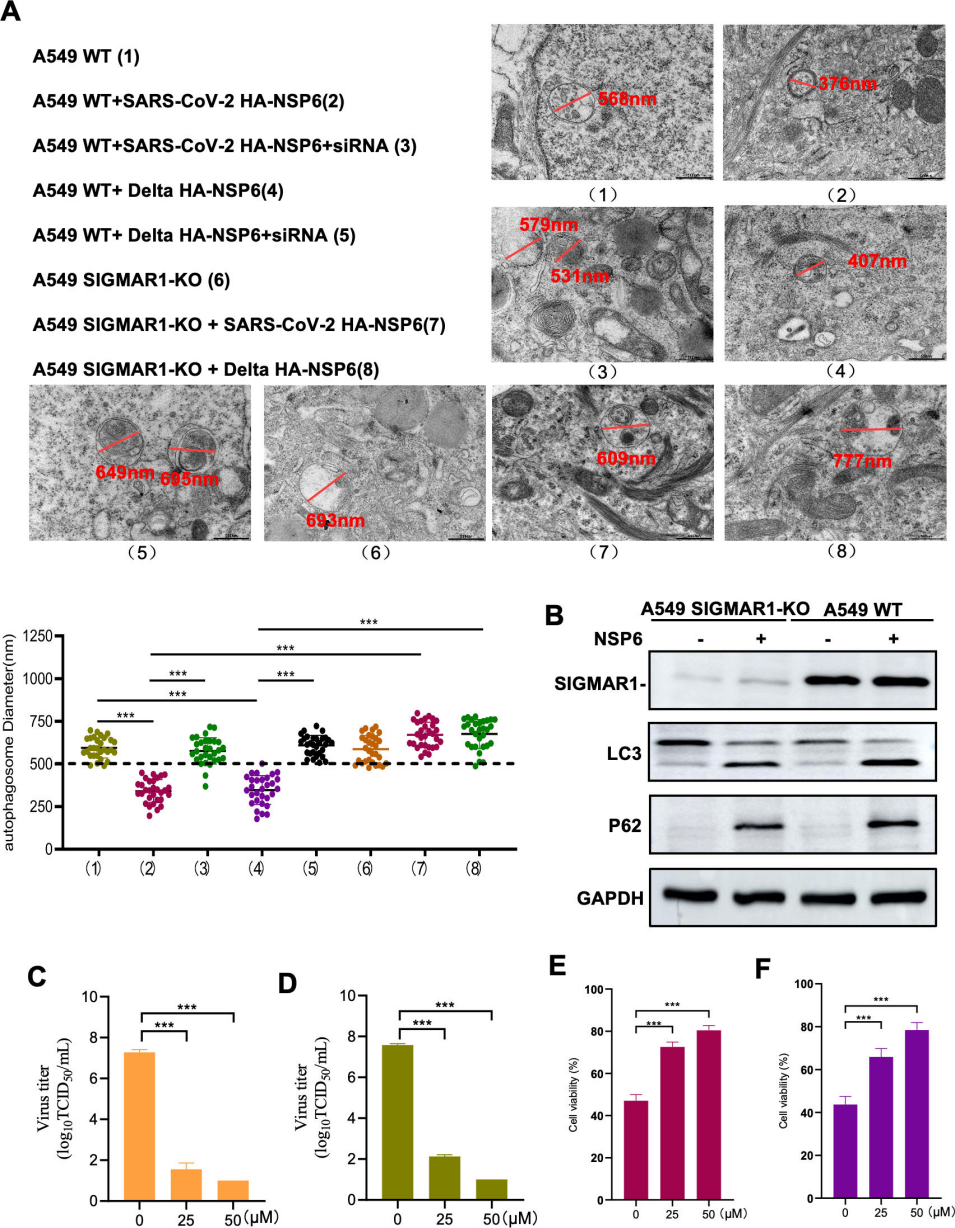
Effect of SIGMAR1 on NSP6-mediated shrinkage of autophagosomes. (**A**) Changes in the diameter of autophagosomes in A549 cells and SIGMAR1-KO A549 cells after transfection with NSP6 were observed using electron microscopy. (**B**) Western blotting showed SIGMAR1, LC3, and P62 protein expression in A549 cells and SIGMAR1-KO A549 cells with HA-NSP6 transfection. (**C and D**) Analysis of the effect of SIGMAR1 inhibitors BD1063 on viral replication of SARS-CoV-2 wild-type strain (C) and the Delta variant (**D**). (**E and F**) Analysis of the effect of BD1063 on the inhibitory effect of SARS-CoV-2 original strain (**E**) and Delta variant (**F**) on Vero E6 cells. ***P < 0.001.

We used TEM to detect the effects of transfection with NSP6-HA of wild-type strain or Delta variant strain of SARS-CoV-2 after SIGMAR1 KO on autophagosomes. The results showed that SIGMAR1 knockout could restore the abnormalities of autophagosome size after transfection with NSP6 (Fig. 6A). Then, we investigated the effects of transfecting NSP6 protein into SIGMAR1-KO cells on LC3 and P62 expression. The results showed that SIGMAR1 KO did not affect the LC3 and P62 expression compared to the WT group after transfection with NSP6 protein (Fig. 6B). This indicates that SIGMAR1 is more responsible for NSP6-induced decreased size of autophagosomes, rather than autophagy flux. A previous study reports that SIGMAR1 inhibitors fluvoxamine, remoxipride, and naltrexone have the potential to treat COVID-19 (22). BD1063, 1-[2-(3,4-dichlorophenyl)ethyl]−4-methylpiperazine dihydroc-hloridee, acts as an antagonist of the sigma-1 receptor and is capable of occupying the binding site of the sigma-1 receptor, thus preventing other ligands (e.g., agonists) from sigma-1 receptors. This occupancy effect blocks the sigma-1 receptor-mediated signaling pathway, which in turn inhibits the physiological function of the sigma-1 receptor, and has been shown to be effective in the treatment of colitis and neurological disorders (23). However, whether it can inhibit virus infection has not been reported. Our results found that there was a significant decrease virus copy number of SARS-CoV-2 original strain and Delta variant in Vero E6 cells (Fig. 6C through F). Meanwhile, the replication of the SARS-CoV-2 in SIGMAR1-KO Vero E6 cells is limited, BD1063 was able to reduce the viral copy number, with little difference in the effect of the two in combination (Fig. S4 and S5).

## DISCUSSION

There is no research indicating the effect of NSP6 of SARS-CoV-2 on autophagosomes. Our results showed that NSP6 of SARS-CoV-2 wild-type strain can promote an increase in autophagosome number and a decrease in volume. Additionally, we determined the impact of NSP6 on the expression of key proteins and phosphorylation levels in the autophagy signaling pathway. Furthermore, we discovered that NSP6 can enhance transcription factor TFEB expression and nuclear translocation, possibly contributing to increased levels of autophagic proteins.

Therefore, we conducted electron microscopy observations on cells transfected with NSP6 of SARS-CoV-2 wild-type strain and other variants. The results showed that only wild-type strain and Delta variant strain could cause a decrease in autophagosome volume. Previous articles have suggested that NSP6 of infectious bronchitis virus (IBV) localizes to the ER and promotes formation of autophagosome structures within ER but does not enter into cytoplasm along with released autophagosomes from ER (24). On the other hand, SARS-CoV NSP6 partially co-localizes with LC3-positive structures, suggesting its possible entry into autophagosomes. It is also speculated that due to shared predicted 5–7 transmembrane topology between all coronaviral NSP6 including IBVs are likely localized at ER membrane as well (25). Our results indicated that SARS-CoV-2 NSP6 localizes to not only in ER membrane but also in lysosomes and can cause lysosomal damage, leading to downregulation of lysosomal membrane-related proteins, as well as upregulation of LC3 and P62. It suggests that SARS-CoV-2 NSP6 has a broader impact on host autophagy, not only in the upstream formation of autophagosomes but also during fusion between autophagosomes and lysosomes.

We further observed the morphology of autophagosomes after transfecting cells with NSP6 of SARS-CoV-2 wild-type strain, Beta variant, Delta variant, and SARS-CoV. The results showed that only NSP6 of SARS-CoV-2 wild-type strain, Delta variant, and SARS-CoV caused a decrease in autophagosome volume while this phenomenon was not observed with Beta variant. This result is consistent with previous research findings which have shown that γ-coronavirus IBV leads to the reduction of autophagosome size (13) and both β-coronaviruses mouse hepatitis virus (MHV) and SARS-CoV can induce autophagy (11, 26). Due to the high similarity between different variants’ NSP6 protein sequences within SARS-CoV-2 family members, it provides convenience for studying how these variations affect the induction of smaller-sized autophagosome structures. Upon sequence comparison, we found that the amino acid expression of the wild-type strain and the Delta variant were identical in the SARS-CoV-2 NSP6. However, the NSP6 of Beta variant and SARS-CoV differs from the above two at three amino acid sequences deletions, 106, 107 and 108 (27). Moreover, some Delta strains contain a T3646A mutation. This may be responsible for the different outcomes.

Since NSP6 can activate mTOR signaling pathway (13, 28), to further determine its effect on autophagosomes, we compared them with rapamycin-treated cells transfected with both mTOR signal activator rapamycin and NSP6. The results showed that after activating the mTOR signaling pathway with rapamycin, there was an increase in autophagosome number but no change in volume. This result suggests that the increase of autophagosomes induced by NSP6 is likely mediated through the mTOR signaling pathway, while factors other than mTOR may be involved in decreasing their volume.

There have been reports predicting the structure of NSP6 and suggesting its potential interaction with SIGMAR1 (14), which plays an important role during ER stress (29). As SIGMAR1 is associated with membrane transport within cells (30), we speculate its involvement in determining autophagosome size. To further investigate how NSP6 induces a decrease in autophagosome volume, we studied the effect of NSP6 through its interaction with SIGMAR1. Our research findings showed that transfection of cells lacking SIGMAR1 (SIGMAR1-KO) with NSP6 does not lead to any changes in autophagosome volume, and Western blot analysis indicates significant decreases in LC3 and P62 expression after SIGMAR1-KO, indicating that the effect of NSP6 on both inducing smaller-sized autophagosome structures as well as the development of autophagy are related to its interaction with SIGMAR1. Additionally, we discovered through drug testing using sigma-1 receptor inhibitors that these inhibitors have antiviral effects by blocking interactions between NSP6 and SIGMAR1.

Finally, we performed proteomic analysis on proteins from WT-transfected cells and SIGMAR1-KO-transfected cells to further study signaling pathways associated with NSP6 protein and SIGMAR1. The results showed that knockdown of SIGMAR1 significantly affected virus defense signal pathways and immune-related pathways, and had impacts on lysosomes. This suggests that SIGMAR1 is not only involved in the process of reduction of autophagosome size induced by NSP6 but also involved in the process of damage of lysosomes.

In conclusion, this study revealed SARS-CoV-2 NSP6 induced autophagosome process. We found that the decrease of autophagosome size mediated by SARS-CoV-2 NSP6 are strongly associated with SIGMAR1 and suggested that SIGMAR1 could be a potential therapeutic target for developing drugs against coronavirus.

## Data Availability

The data sets used and analyzed during the current study are available from the corresponding author on reasonable request.

## SUPPLEMENTAL MATERIAL

Supplemental figures jvi.00754-24-s0001.pdf Figures S1 to S5.

ASM does not own the copyrights to Supplemental Material that may be linked to, or accessed through, an article. The authors have granted ASM a non-exclusive, world-wide license to publish the Supplemental Material files. Please contact the corresponding author directly for reuse.
