# Supplementary material for: SARS-CoV-2 NSP6 reduces autophagosome size and affects viral replication via sigma-1 receptor
Source: J Virol. 2024 Oct 24;98(11):e00754-24. doi: 10.1128/jvi.00754-24 (PMC11575221; doi:10.1128/jvi.00754-24)
Supplement: Supplemental figures — Figures S1 to S5. [file jvi.00754-24-s0001.pdf]

Supplementary information

SARS-CoV-2 NSP6 reduces autophagosome size and affects viral replication via Sigma-1 Receptor

Cuiling Zhang[a], Qiwei Jiang[a], Zirui Liu[a], Nan Li[a], Zhuo Hao[a], Gaojie Song[d], Dapeng Li[c], Minghua chen[a], Lisen Lin[e], Yan Liu[a], Xiao Li[a,b #], Chao Shang[a#] and Yiquan Li[b#]

[a] Changchun Veterinary Research Institute, Chinese Academy of Agricultural Sciences, Changchun 130122, P. R. China.

[b] Changchun University of Chinese Medicine, Changchun, P.R. China

[c] Department of Neurosurgery, First Hospital of Jilin University, Changchun, 130021, P.R. China.

[d] Jiangxi Provincial Key Laboratory of Systems Biomedicine, Jiujiang University, Jiujiang, China.

[e] Department of Chemistry, Northeastern University, Shenyang, 110819, P. R. China.

Supplementary figures

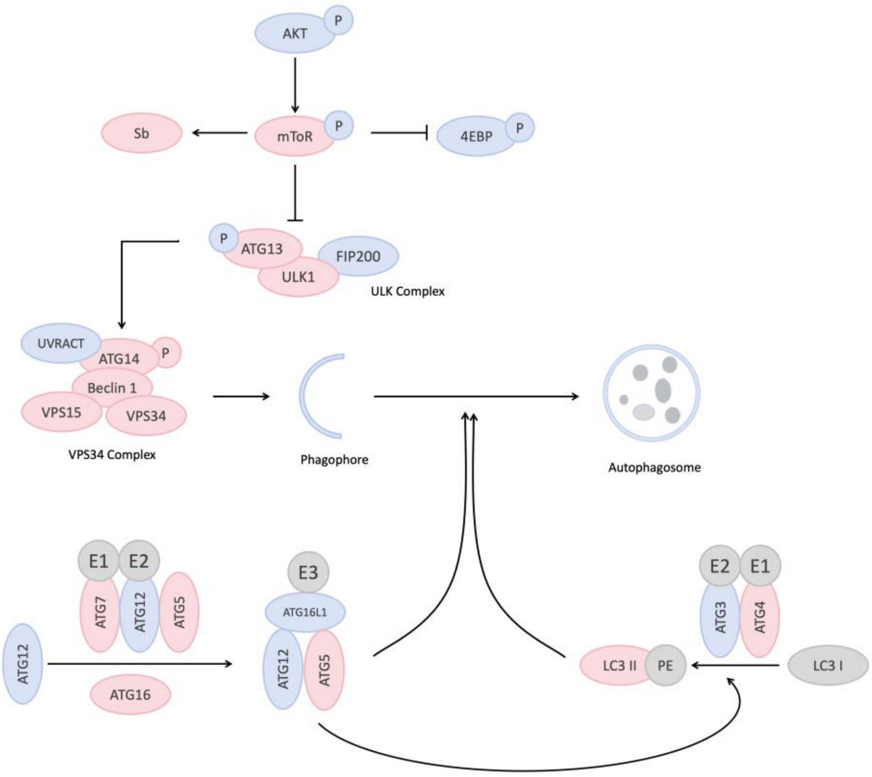

Figure S1. Pathway diagram of autophagy-related proteins.Red represents genes upregulated after nsp6 treatment, blue represents downregulated genes, arrows indicate promotion, and the other line indicates inhibition.

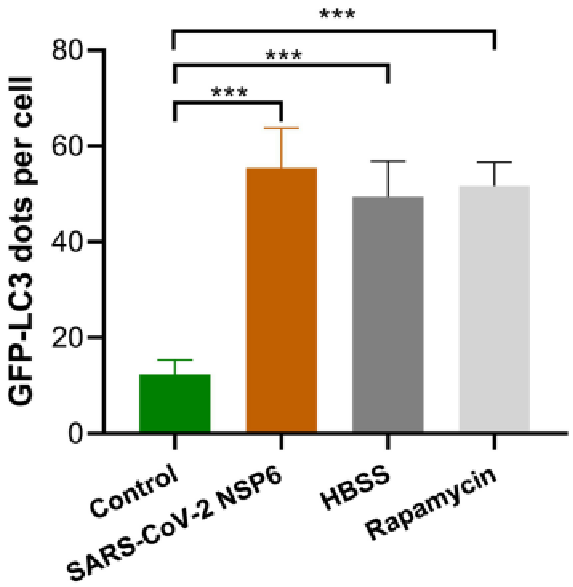

Figure S2. Analysis the number of autophagosomes in A549 cells after NSP6 transfection, rapamycin treatment, or starvation were observed using confocal microscopy. ******p < 0.001*.**

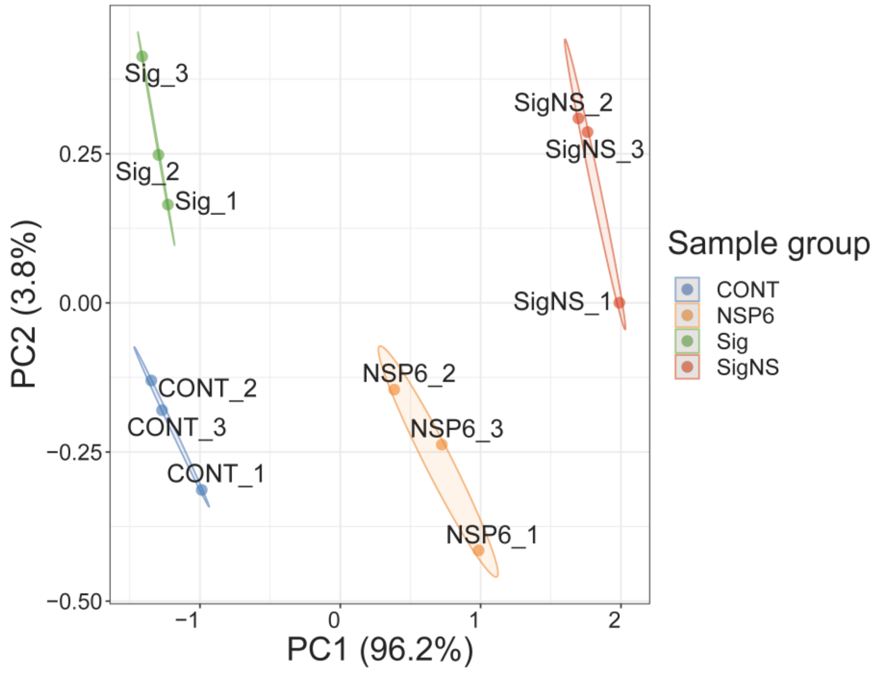

Figure S3. PCA analysis between four groups (NSP6: HA-nsp6 A549 WT; CONT: A549 WT; SigNS: HA-nsp6 A549 SIGMAR1 KO; Sig: A549 SIGMAR1 KO).

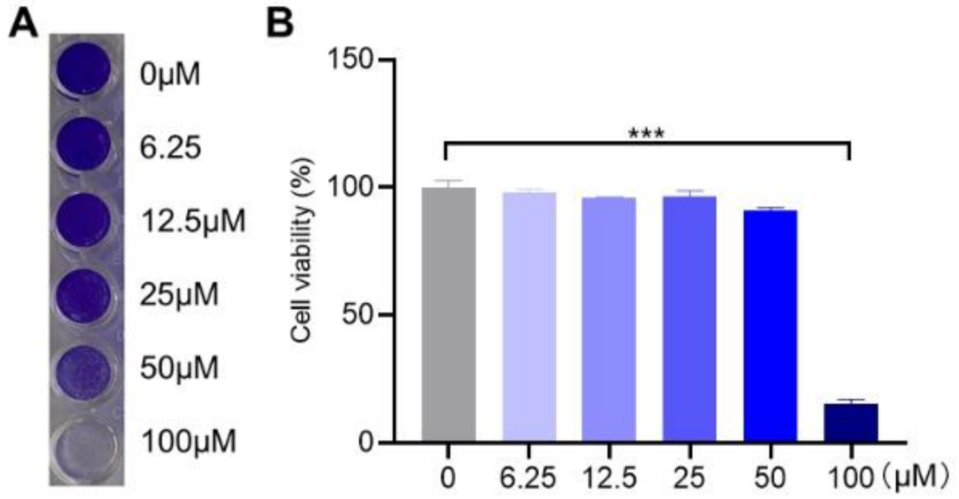

Figure S4. Analysis of cytotoxic effects of BD1063 on Vero E6 cells at different concentrations using crystal violet staining (A) and CCK-8 assay (B). ******p < 0.001*.**

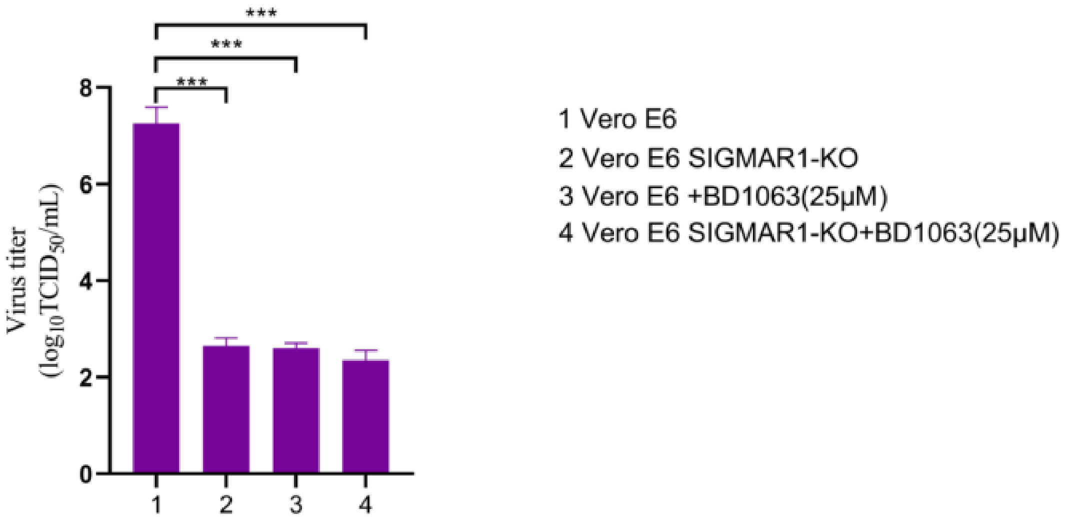

Figure S5. Analysis of antiviral effects of BD1063 on Vero E6 or SIGMAR1-KO cells. ******p < 0.001*.**
